# Chloroplast Retrograde Regulation of Heat Stress Responses in Plants

**DOI:** 10.3389/fpls.2016.00398

**Published:** 2016-03-31

**Authors:** Ai-Zhen Sun, Fang-Qing Guo

**Affiliations:** The National Key Laboratory of Plant Molecular Genetics, National Center of Plant Gene Research (Shanghai) and CAS Center for Excellence in Molecular Plant Sciences, Institute of Plant Physiology & Ecology, Shanghai Institutes for Biological Sciences, Chinese Academy of SciencesShanghai, China

**Keywords:** chloroplast, retrograde signaling, heat stress responses, plastid signal molecules, reactive oxygen species (ROS)

## Abstract

It is well known that intracellular signaling from chloroplast to nucleus plays a vital role in stress responses to survive environmental perturbations. The chloroplasts were proposed as sensors to heat stress since components of the photosynthetic apparatus housed in the chloroplast are the major targets of thermal damage in plants. Thus, communicating subcellular perturbations to the nucleus is critical during exposure to extreme environmental conditions such as heat stress. By coordinating expression of stress specific nuclear genes essential for adaptive responses to hostile environment, plants optimize different cell functions and activate acclimation responses through retrograde signaling pathways. The efficient communication between plastids and the nucleus is highly required for such diverse metabolic and biosynthetic functions during adaptation processes to environmental stresses. In recent years, several putative retrograde signals released from plastids that regulate nuclear genes have been identified and signaling pathways have been proposed. In this review, we provide an update on retrograde signals derived from tetrapyrroles, carotenoids, reactive oxygen species (ROS) and organellar gene expression (OGE) in the context of heat stress responses and address their roles in retrograde regulation of heat-responsive gene expression, systemic acquired acclimation, and cellular coordination in plants.

## Introduction

Taking into account the presence of genes encoding organellar proteins in different cellular compartments of the plant cell, intracellular communication is critical for regulation and coordination of a variety of physiological processes, including responses to biotic and abiotic stresses. By definition, retrograde signaling is a communication pathway whereby the transcriptional activities in the nucleus are regulated in part by signals derived from plastids and mitochondria. According to existing literatures, two categories can be largely classified in respect to retrograde signaling, including developmental control of organelle biogenesis, and operational control to adjust and acclimate to environmental stresses (Pogson et al., 2008). In general, the chloroplasts in plants and algae are assumed to be the descendants of the ancient photosynthetic bacteria. As an semi-autonomous organelle, the chloroplast maintains a similar circular genome and transcription and translation machinery like its evolutionary precursor (Watson and Surzycki, 1983; Sugiura et al., 1998). Because the components of the complex energetic reactions linked to photosynthesis are encoded by organelle and nuclear genomes, gene expression in these separate compartments require the existence of sophisticated regulatory mechanisms that ensure adequate synthesis of proteins functioning in common photosynthetic complexes. The tightly coordinated gene expression in both nucleus and chloroplast is required for the correct stoichiometric subunit composition of these complexes. It is generally accepted that anterograde signals originating from the nucleus and retrograde signals emerging from the chloroplast orchestrate this intracellular coordination (Woodson and Chory, 2008).

Historically, the first report describing the retrograde signaling was based on two barley chloroplast ribosome-deficient mutants, the barley (*Hordeum vulgare*) *albostrians*, whose defects in plastid functions result in downregulation of nuclear-encoded plastid proteins (Bradbeer et al., 1979). Since, this revolutionary discovery, the intensive studies have been focusing on the function of retrograde signaling in plastid development by coordinating chlorophyll biosynthesis with the expression of nuclear genes that encode plastid-localized chlorophyll-binding proteins using young seedlings of mustard, *Arabidopsis*, pea, or barley with lincomycin, chloramphenicol, or streptomycin, the inhibitors of plastid protein synthesis (Oelmuller et al., 1986; Susek et al., 1993; Yoshida et al., 1998; Sullivan and Gray, 1999). The *gun* (genome uncoupled) mutants where the communication between the chloroplast and the nucleus has been disrupted are very helpful in deciphering the retrograde signaling phenomenon (Woodson and Chory, 2008; Barajas-Lopez et al., 2013; Chi et al., 2013). The mutant seedlings express nuclear-encoded photosynthetic genes (PhANG) despite defective chloroplast physiology or inhibited biogenesis (Susek et al., 1993). According to restrictions in defined steps in tetrapyrrole biosynthesis, identification of the *gun*2, *gun3*, *gun4*, and *gun5* mutants provided evidence that accumulation of the chlorophyll intermediate Mg-protoporphyrin IX (Mg-Proto IX) is involved in initiation of retrograde signaling whereas the *gun1* mutant results from mutation in a gene encoding a chloroplast-localized pentatricopeptide repeat-containing protein (PPR; Susek et al., 1993; Mochizuki et al., 2001; Larkin et al., 2003; Strand et al., 2003; Koussevitzky et al., 2007).

The involvement of the key enzymes of the tetrapyrrole biosynthesis pathway in the *gun* phenotype led to numerous studies on tetrapyrroles, especially Mg-protoporphyrin IX (Mg-ProtoIX), as putative retrograde signals. Large changes in nuclear gene expression have been triggered by stress-induced perturbations of tetrapyrrole biosynthesis and specific accumulation of the chlorophyll biosynthetic intermediate Mg-ProtoIX and its methylester (Mg-ProtoIX-ME) have been shown to coincide with these changes in nuclear gene expression (Johanningmeier and Howell, 1984; Kropat et al., 1997, 2000; Strand et al., 2003; Alawady and Grimm, 2005; Pontier et al., 2007; von Gromoff et al., 2008). By taking advantage of the fluorescent properties of tetrapyrroles, Mg-ProtoIX could be visualized to accumulate both in the chloroplasts and the cytosol under stress conditions using confocal laser scanning spectroscopy (Ankele et al., 2007), suggesting a possibility that the signaling metabolite Mg-ProtoIX is exported from the chloroplast to the cytosol, thus to transmit the plastid signal to nucleus. However, the role of Mg-ProtoIX/Mg-ProtoIX-ME as a plastid signal was subsequently questioned because no correlation between the accumulation of Mg-ProtoIX and retrograde signaling was observed in two different studies (Mochizuki et al., 2008; Moulin et al., 2008). Given that Mg-ProtoIX is phototoxic, its accumulation within cytosol might induce problematic effects on cellular homeostasis. Thus, the identity of retrograde signals remains worthy of further investigation. Interestingly, a recent report identified heme as a strong candidate for mediating chloroplast-to-nucleus signaling (Woodson et al., 2011). Two plastid-derived isoprenoid derivatives, methylerythritol cyclodiphosphate, and β-cyclocitral, were reported to function as retrograde signal molecules although their receptor(s) or sites of action in the nucleus remain largely unknown (Ramel et al., 2012; Xiao et al., 2012). Importantly, as an intermediate of the methylerythritol phosphate (MEP) pathway, methylerythritol cyclodiphosphate accumulates in response to abiotic stresses (Xiao et al., 2012). Similarly, β-Cyclocitral, a volatile apocarotenoid derived from β-carotene, accumulates in response to singlet oxygen (^1^O_2_) and light stresses and regulates nuclear gene expression (Ramel et al., 2012, 2013). Moreover, ^1^O_2_-induced signaling and feedback was proposed to mediate the impact of tetrapyrrole biosynthesis on nuclear gene expression (Schlicke et al., 2014). These findings indicate that the nature of retrograde signaling remains largely undefined.

Plants have evolved complex signaling networks to sense and respond to environmental stresses. It has been assumed that chloroplasts act as a specific sensor of intra- and extracellular stimuli and can integrate a multitude of intracellular signals and pathways in order to sustain homeostasis at both the cellular and organismal levels. With respect to chloroplast-nuclear signaling in response to environmental stimuli, intensive studies have been focusing on the initiation of signaling cascades in the chloroplast and transcriptional changes in the nucleus. In the past few years, a number of retrograde signaling pathways were identified and/or proposed as stress-specific organelles-to-nucleus retrograde signaling cascades. This review focuses on the recent advancements in revealing the order of events that induce the activation of the acclimation response to abiotic stresses. Special attention was given to the retrograde regulatory networks identified in studies on cellular responses to heat stress.

## Retrograde Signaling Pathways in Heat Stress Responses

According to existing literatures, a temperature upshift, usually 10–15°C above an optimum temperature for growth, is taken as heat stress for photosynthetic processes in higher plants (Wahid et al., 2007; Allakhverdiev et al., 2008). Considering that components of the photosynthetic apparatus in the chloroplast are susceptible targets of thermal damage in plants, the chloroplasts were proposed as sensors to heat stress. It should be noted that heat stress commonly causes severe thermal damages to photosystem II (PSII), the most heat-sensitive photosynthetic apparatus within the chloroplast thylakoid membrane protein complexes involved in photosynthetic electron transfer and ATP synthesis (Berry and Bjorkman, 1980; Havaux, 1993; Sharkey, 2005; Wahid et al., 2007; Allakhverdiev et al., 2008). As common indicators of heat stress-induced damages, chlorophyll fluorescence, the ratio of variable fluorescence to maximum fluorescence (*F*_v_/*F*_m_) and the base fluorescence (*F*_o_), correlates with disruptions of photochemical reactions in thylakoid lamellae of chloroplasts (Yamada et al., 1996; Wise et al., 2004; Wahid et al., 2007; Allakhverdiev et al., 2008). Particularly, heat stress causes the dissociation of oxygen evolving complex (OEC) in PSII, which further results in an imbalance in the electron flow from OEC toward the acceptor side of PSII in the direction of PSI reaction center (Havaux and Tardy, 1996; Klimov et al., 1997; Wahid et al., 2007; Allakhverdiev et al., 2008). Moreover, the reaction center-binding protein D1 of PSII was cleaved and a manganese (Mn)-stabilizing 33-kDa proteins was dissociated from PSII reaction center complex in spinach thylakoids upon heat stress treatment (Yamane et al., 1997). In addition to damages on OEC in PSII, heat stress also causes dramatic reduction in the efficiency in carbon assimilation metabolism in the stroma of chloroplast (Sharkey, 2005).

In the prokaryotic and eukaryotic kingdoms, the heat stress response as a universal cellular response represents the first line of inducible defense against imbalances in cellular homeostasis. In higher plants, a rapid expression reprogramming of a set of proteins known as heat shock proteins (HSPs) is considered to be a marked activation of heat stress response (Kotak et al., 2007; von Koskull-Doring et al., 2007). Based on the analysis of *Arabidopsis* and crop plants genome-wide expression profiles, the transcripts of the well-characterized *HSPs* increased dramatically, including *Hsp101*, *Hsp70s*, and small *HSPs*, which are proposed to act as molecular chaperones in protein quality control under heat stress (Rizhsky et al., 2004; Busch et al., 2005; Lim et al., 2006; Schramm et al., 2006; Larkindale and Vierling, 2008; Matsumoto et al., 2014; Wang et al., 2014b; Frey et al., 2015).

In the unicellular green alga *Chlamydomonas reinhardtii*, Mg-ProtoIX and Mg-ProtoIX-ME were previously shown to transiently induce the expression of *HEAT SHOCK PROTEIN 70A/B* (*HSP70A* and *HSP70B*) encoding the cytosolic and the plastid-localized proteins, respectively (Kropat et al., 1997, 2000). However, the light-induction of HSP70 is impaired in the chlorophyll-deficient *brs-1* mutant, indicating that the accumulation of Mg-ProtoIX is essential for the light induction of HSP70 (Hess et al., 1994). Using the four *Chlamydomonas reinhardtii* mutants in the Mg-chelatase that catalyzes the insertion of magnesium into protoporphyrin IX, the reduced levels of Mg-tetrapyrroles but increased levels of soluble heme were detected in the four mutants and the light-induction of HSP70A was preserved, although Mg-ProtoIX has been implicated in this induction (von Gromoff et al., 2008). More importantly, *HSP70A* was activated by feeding Hemin to algae cultures in the dark and this induction was mediated by the same plastid response element (PRE) in the *HSP70A* promoter that has also been shown to mediate induction by Mg-ProtoIX and light. Such communication likely involves both Hemin and Mg-ProtoIX, respectively, indicating that these signals converge on the same pathway (von Gromoff et al., 2008). Studies focused on the induction specificity of these two tetrapyrroles demonstrated that neither Proto, nor Pchlide or Chlide was able to induce the nuclear genes (Kropat et al., 1997). A model was derived according to accumulated literature (Beck, 2001, 2005): MgProto and/or MgProtoMe, produced in the plastid become(s) accessible on the cytoplasmic side of the chloroplast after light activation of the *HSP70* genes within the chloroplast or its envelope. In cytoplasm or nucleus, putative factor(s) may recognize these tetrapyrroles, either regulating expression of the nuclear HSP70 genes directly or stimulating a signaling cascade that enters the nucleus.

Given that the tetrapyrroles Mg-ProtoIX and heme have been implicated in the retrograde control of nuclear gene expression in *Chlamydomonas reinhardtii*, the intensive studies have been focusing on genome-wide transcriptional profiling to explore the global impact of these tetrapyrroles on regulation of gene expression and the scope of the response. Upon feeding with Mg-ProtoIX and heme, almost 1,000 genes were shown to be changed transiently but significantly (Voss et al., 2011). Most of these genes encoded enzymes of the tricarboxylic acid cycle, heme-binding proteins, stress-response proteins, as well as proteins involved in protein folding and degradation whereas only a few genes were involved in photosynthetic processes. More than 50% of the latter class of genes was also regulated by heat shock. Significantly, 51% of the 982 tetrapyrrole-regulated genes were also activated in response to heat stress, indicating that both tetrapyrroles function as secondary messengers for adaptive physiological responses affecting the entire cell and not only organellar proteins.

The chloroplast in plants and algae maintains a circular genome and transcription and translation machinery similar to that of its evolutionary precursor, the descendants of the ancient photosynthetic bacteria (Sugiura et al., 1998). Accordingly, the majority of chloroplast proteins encoded in the nucleus are imported into the chloroplasts after biosynthesis in the cytoplasm (Jarvis, 2008). The existence of an organellar gene expression (OGE)-dependent retrograde signal pathway was first suggested more than 30 years ago (Bradbeer et al., 1979). Subsequently, numerous studies found that treatments with inhibitors of OGE, such as chloramphenicol, lincomycin, or erythromycin severely inhibited the expression of nuclear genes for photosynthesis-related proteins during early stages of plastid development (Oelmuller et al., 1986; Susek et al., 1993; Yoshida et al., 1998; Sullivan and Gray, 1999). One of working modules for the OGE-dependent retrograde signal pathway is that a plastid-localized pentatricopeptide repeat (PPR) protein, encoded by *GUN1*, integrates the signal cascades triggered by the aberrant plastid functions before the integrated signal is transmitted into the nucleus by a regulatory mechanism involving the transcription factor ABA INSENSITIVE4 (ABI4; Koussevitzky et al., 2007). It has been proposed that a disruption on protein synthesis in the chloroplast could give rise to a signal or that the affected plastids keep away from the stage at which they could send the appropriate signal while the identity of the components of the signaling cascade and, in particular, the primary target genes and transcriptional factors (TFs) mediating this response remain largely unknown (Kleine et al., 2009). A recent report revealed that the chloroplast translational capacity is a critical factor in generating the retrograde signal (s) to activate the heat-responsive expressions of the heat stress transcription factor *HsfA2* and its target genes (Yu et al., 2012). In the study described above, the chloroplast ribosomal protein S1 (RPS1) was identified as a heat-responsive protein, functioning as a subunit protein of the plastid ribosome in synthesis of photosynthetic proteins in *Arabidopsis*. In general, mutations of *RPS1* causes the translational defects in chloroplasts repress the nuclear heat-responsive gene expression upon heat treatments, revealing a novel regulatory mechanism whereby plant cells trigger heat-responsive activation of the nuclear gene expression to keep accordance with the current status of chloroplasts under heat stress.

Accumulated evidence has also shed light on the effect of chloroplast–mitochondria signaling interactions on stress responses. The tight communication between chloroplasts and mitochondria is required for balancing the activities of the two energy organelles under normal growth conditions or in adaptation to environmental stresses (Woodson and Chory, 2008; Blanco et al., 2014; Ng et al., 2014; Allahverdiyeva et al., 2015; Bobik and Burch-Smith, 2015; Gollan et al., 2015; Van Akent and Van Breusegem, 2015). It has been proposed that the metabolite exchange between the organelles via translocators located on envelope membranes of chloroplasts and mitochondria acts as an important channel of communication, which contributes to their central roles in energy capture and utilization (Blanco et al., 2014; Bobik and Burch-Smith, 2015). As for the intra-mitochondrial stress response, energy-dissipating components modulate the retrograde signaling pathways from mitochondria, which controls the cellular adaptation processes under stress conditions (Ng et al., 2014; Rurek, 2014). In *Arabidopsis*, AOX1a isoform is induced by heat stress (Elhafez et al., 2006) and seems to be also regulated by chloroplasts upon highlight treatment (Finnegan et al., 1997; Blanco et al., 2014). Recent studies suggest that AOX1a functions in optimizing photosynthesis by sustaining the chloroplastic redox state and regulating cellular redox homeostasis when electron transport through the COX pathway is disturbed at complex III (Pu et al., 2015; Vishwakarma et al., 2015). Interestingly, ABI4 was shown to regulate the responsive expression of both *Lhcb* and *AOX1A* genes, suggesting that ABI4 acts as a critical molecular link for coordinating the communication between chloroplasts and mitochondria (Koussevitzky et al., 2007; Giraud et al., 2009). A recent report suggests that the nuclear localized cyclin-dependent kinase E1 (CDKE1), a prerequisite for *AOX* induction, acts as a central nuclear component integrating mitochondrial and plastid retrograde signals, which could modulate energy metabolism under stress (Blanco et al., 2014). The mitochondrial retrograde signaling and auxin signaling are reported to be reciprocally regulated in balancing growth and environmental stresses (Ivanova et al., 2014) and a membrane-bound NAC transcription factor, ANAC017, was identified to mediate mitochondrial retrograde signaling by genetic screening regulators of alternative oxidase1a mutants in *Arabidopsis* (Ng et al., 2013). It has been known that mitochondria of plant cells modulate the cytosolic Ca^2+^ level by changing the potential at the inner mitochondrial membrane, functioning in the retrograde regulation of expression of heat-responsive genes (Pyatrikas et al., 2014; Rikhvanov et al., 2014). However, delineating these stress-interacting networks between chloroplasts and mitochondria involving the perception and integration of stress stimuli is important and most of network components remain largely unexplored.

Based on an update on recent findings related to plastid-to-nucleus metabolic signals in plants, it has been suggested that the metabolic reprogramming under a variety of environmental stresses relates to the functional alteration of essential cell compartments, such as chloroplasts (Estavillo et al., 2011, 2013; Xiao et al., 2012, 2013; Chi et al., 2015). Plastid retrograde signals could be generated from various sources, including the tetrapyrrole pathway, the level of ROS, or the related metabolic processes in the plastids as described in **Table 1**. Particularly, ROS among all these signals are thought to play a key role in modulating initial signal cascades in higher plants. Recently, new plastid metabolic signals within chloroplasts have been identified in *Arabidopsis* (Estavillo et al., 2013; Xiao et al., 2013; Chi et al., 2015), emphasizing the role of plastids in abiotic-stress sensing and signaling in a retrograde way.

**Table 1:** The reported signal molecules involved in plastid-to-nucleus retrograde pathways.

	Plant system	Related abiotic stresses and physiological processes	Reference
**Signal molecules**			
Mg-ProtoIX Mg-ProtoIX-ME	*Arabidopsis*	Expression of nuclear-encoded photosynthetic genes (PhANG)	Strand et al., 2003
Heme	*Arabidopsis*	Expression of nuclear-encoded PhANG	Woodson et al., 2011
Mg-ProtoIX Mg-ProtoIX-ME	*Chlamydomonas reinhardtii*	Induction of *HSP70* expression	Hess et al., 1994; Kropat et al., 1997
Hemin	*Chlamydomonas reinhardtii*	Induction of *HSP70* expression	von Gromoff et al., 2008
MEcPP	*Arabidopsis*	Oxidative stress	Xiao et al., 2012
β-Cyclocitral	*Arabidopsis*	Singlet oxygen (^1^O_2_) and light stress	Ramel et al., 2012
Methylerythritol cyclodiphosphate	*Arabidopsis*	Singlet oxygen (^1^O_2_) and light stress	Ramel et al., 2012
**Reactive oxygen species (ROS)**			
Hydrogen peroxide Superoxide radicals	*Nicotiana tabacum*	Heat stress	Vacca et al., 2006; Wang et al., 2006
Hydrogen peroxide	*Arabidopsis*	Heat stress and oxidative stress	Nishizawa et al., 2006
	Tomato	Heat stress	Banzet et al., 1998
	Rice	Heat stress and oxidative stress	Lee et al., 2000
Singlet oxygen Hydroxyl radicals	Spinach	Heat stress	Yamashita et al., 2008
Singlet oxygen	*Arabidopsis*	Heat stress	Lin et al., 2014
Singlet oxygen	*Arabidopsis*	Oxidative stress	Wagner et al., 2004
Nitric oxide (NO)	*Arabidopsis*	Heat stress	Wang L. et al., 2014
PAP	*Arabidopsis*	High light and drought stresses	Estavillo et al., 2011


## ROS as Retrograde Signals in Heat Stress Responses

As an unavoidable consequence of aerobic metabolism, plants permanently produce a variety of reactive oxygen species (ROS) such as hydrogen peroxide (H_2_O_2_), superoxide, hydroxyl radicals (^⋅^OH), and ^1^O_2_. It is well known that the chloroplast is a major producer of ROS during photosynthesis and contains a large array of ROS-scavenging mechanisms (Asada, 2006). A variety of abiotic stresses inhibit the excitation energy transfer in the PSII antenna complex and the electron transport in the PSII reaction center in algae and higher plants and the limitation in the excitation energy transfer and the electron transport is accompanied with the formation of ROS (Suzuki et al., 2012). ROS such as ^1^O_2_ is formed by the excitation energy transfer, whereas superoxide anion radical (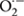), H_2_O_2_ and ^⋅^OH are formed by the electron transport (Pospisil and Prasad, 2014). ROS are proposed to diffuse away from their sites of production and consequently elicit a different set of signaling events under a wide range of biotic and abiotic stress conditions (Neill et al., 2002; Mittler et al., 2004; Miller et al., 2008; Suzuki et al., 2012). A variety of operational retrograde signaling pathways are thought to be triggered by ROS and photosynthesis redox imbalance during stress conditions and play an important role in the acclimation of plants (Pogson et al., 2008; Galvez-Valdivieso and Mullineaux, 2010; Suzuki et al., 2012). On the other hand, the current redox-status of chloroplasts, which correlates with the imbalance of ROS accumulation caused by abiotic stresses, may be transmitted by monitoring the state of the plastoquinone, ascorbate, and glutathione pools (Foyer et al., 2009; Suzuki et al., 2012; Foyer and Noctor, 2013; Petrov et al., 2015). In addition to chloroplasts, several other heat stress-dependent ROS production sites have been described. It has been suggested that the respiratory burst oxidase homolog D (RBOHD), acting as a ROS-generating NADPH oxidase in the plasma membrane, could function in the oxidative burst occurring during heat stress (Suzuki et al., 2011). The accumulation of H_2_O_2_ can be inhibited by an inhibitor of the enzyme NADPH oxidase in *Arabidopsis* and tobacco cell cultures, suggesting that RBOHD has a central role in heat stress signaling and thermotolerance (Larkindale et al., 2005; Volkov et al., 2006; Koenigshofer et al., 2008; Miller et al., 2009). It was reported that an impaired mitochondrial metabolism seems to be responsible for oxidative bursts occurring in tobacco cells undergoing heat-induced programmed cell death (PCD), suggesting that production of ROS during heat stress can also occur in mitochondria (Vacca et al., 2004; Valenti et al., 2007). Recent studies suggest that AOX1A plays a significant role in regulating ROS generation when electron transport is disrupted through the COX pathway (Vishwakarma et al., 2015). It was reported that the protonophore CCCP-induced depolarization of the mitochondrial membrane inhibited ROS generation upon heat treatments, suggesting that heat stress-induced mitochondrial membrane hyperpolarization causes the increased ROS production in plant cells (Fedyaeva et al., 2014). As a mitochondrial inner membrane protein, uncoupling protein one (UCP1) is able to uncouple the electrochemical gradient from adenosine-5′-triphosphate (ATP) synthesis, dissipating energy as heat. Overexpressing a plant UCP1 ortholog in the mitochondrial inner membrane triggered increased uncoupling respiration and inhibited ROS accumulation under abiotic stresses (Barreto et al., 2014). As a key phospholipid in mitochondrial membranes, cardiolipin (CL) is involved in maintaining the functional integrity and dynamics of mitochondria. The mutations of CARDIOLIPIN SYNTHASE (CLS) caused defects in mitochondrial morphogenesis and stress response to heat treatments in *Arabidopsis*, which has revealed a novel regulatory mechanism in adaptation to environmental stresses in plants (Pan et al., 2014). According to the plant mitochondrial literatures, a prominent theme is likely to link mitochondrial composition to environmental stress responses (Miller et al., 2008; Mittler et al., 2011; Jacoby et al., 2012; Suzuki et al., 2012; Noctor et al., 2014; Van Akent and Van Breusegem, 2015; You and Chan, 2015). Particularly, peroxisomes are another site of ROS generation under conditions that increase photorespiration, such as stomatal closure (Vanderauwera et al., 2011).

As the organelles in which photosynthesis occurs, chloroplasts are extremely susceptible to heat stress (Yamada et al., 1996; Wise et al., 2004; Wahid et al., 2007; Allakhverdiev et al., 2008). Several lines of evidence suggest that different environmental stresses, including also high temperature, can result in oxidative bursts of superoxide and/or hydrogen peroxide in plants (Foyer et al., 1997; Dat et al., 1998; Vallelian-Bindschedler et al., 1998). Exposure to high temperature stress can lead to the increased accumulation of ROS in PSI, PSII as well as in the Calvin–Benson cycle, which cause irreversible oxidative damage to cells (Asada, 2006; Suzuki et al., 2012). ROS that are produced in chloroplasts can function as plastid signals to inform the nucleus to activate the expression of genes encoding antioxidant enzyme and to adjust the stress-responsive machinery for more efficient adaptation to environmental stresses. Under high temperature conditions, tobacco cells produce large amounts of ROS, which are a prerequisite for triggering PCD signaling cascades since application of the antioxidants ascorbate or superoxide dismutase (SOD) to the cultures supports cell survival (Vacca et al., 2006). Upon heat stress treatment, hydrogen peroxide accumulated in the leaves of tobacco (*Nicotiana tabacum*) defective in ndhC–ndhK–ndhJ (Delta ndhCKJ), suggesting the function of the NAD(P) H dehydrogenase-dependent pathway in suppressing the accumulation of ROS in chloroplasts (Wang et al., 2006). These results also indicate that the cyclic photophosphorylation via the NDH pathway might play an important role in regulation of CO_2_ assimilation under heat-stressed condition, thus optimizing the photosynthetic electron transport and reducing the generation of ROS. Under moderate heat treatment conditions, cleavage of the reaction center-binding D1 protein of photosystem II was observed in spinach thylakoid membranes (Yamashita et al., 2008). In accordance with this, ^1^O_2_ and ^⋅^OH were detected in spinach PSII membranes, suggesting that the ROS are generated by heat-induced inactivation of a water-oxidizing manganese complex and through lipid peroxidation. In *Arabidopsis*, following heat treatment, the chlorophyll synthase mutant (*chlg-1*) accumulated a substantial level of chlorophyllide a, which resulted in a surge of phototoxic singlet oxygen, suggesting that chlorophyll synthase acts in maintenance of ROS homeostasis in response to heat stress (Lin et al., 2014).

Unlike plastid gene expression (PGE)-dependent signals, the ROS-dependent retrograde signaling pathways are thought to be primarily acting in adaptation to environmental stresses rather than genome coordination (Woodson and Chory, 2008). Among a variety of ROS-dependent retrograde signaling pathways, most of studies are focusing on the singlet oxygen pathway, which is independent of Mg-ProtoIX and GUN1-mediated signaling (Suzuki et al., 2012). Unlike H_2_O_2_, ^1^O_2_ is a highly reactive radical that is involved in signaling pathway leading to cell death or to acclimation (Wagner et al., 2004). Using the conditional flu mutants that accumulate protochlorophyllide, a potent photosensitizer and generate large amounts of ^1^O_2_ during dark adaptation and upon re-exposure to light, the singlet oxygen signaling pathway has been extensively studied in *Arabidopsis* (Meskauskiene et al., 2001; op den Camp et al., 2003; Apel and Hirt, 2004; Wagner et al., 2004; Laloi et al., 2007; Lee et al., 2007). Importantly, the accumulated ^1^O_2_ in the *flu* chloroplasts correlates with the induction of stress responses, including dramatic alterations in nuclear gene expression and enhanced biosynthesis of the stress hormones, SA, Et, and JA (op den Camp et al., 2003). Moreover, these ^1^O_2_-induced changes were regulated by the chloroplastic proteins EXECUTER1 (EX1) and EXECUTER 2 (EX2) through a distinct pathway (Wagner et al., 2004; Lee et al., 2007; Kim et al., 2012). Interestingly, D1 protein has been identified as a primary target of ^1^O_2_ and seems to act as a major scavenger of ^1^O_2_ because of being close to the site of ^1^O_2_ formation in the RC of PSII (Vass and Cser, 2009). D1 protein is well known for its high turnover rate, which has been attributed to the rapid degradation of the oxidized D1 protein after its interaction with ^1^O_2_ and its replacement by newly synthesized D1 polypeptides (Aro et al., 1993; Lindahl et al., 2000). In addition to the D1 protein, β-carotene, plastoquinol, and α-tocopherol have also been identified as scavenger of ^1^O_2_ and protect PSII against photo-oxidative damage (Telfer et al., 1994; Kruk and Trebst, 2007). Given that the half-life of ^1^O_2_ is very short (200 ns), it is unlikely to escape from the plastid compartment and transmit to nucleus. Thus, more stable second messengers derived from ^1^O_2_ within the plastid are assumed to activate a signaling pathway in controlling the expression of nuclear genes. As one of the main ^1^O_2_ quenchers in chloroplasts, β-carotene can be oxidized by ^1^O_2_ to produce β-cyclocitral that was found to be involved in singlet oxygen-dependent retrograde signaling (Ramel et al., 2012).

As the most stable ROS, H_2_O_2_ serves as a signaling molecule that plays a crucial role in environmental stress responses (Mittler et al., 2011; Suzuki et al., 2012). To survive under heat stress conditions, plants undergo a process of stress acclimation in which HSP expression is promptly activated by specific heat shock transcription factors (HSFs) binding the conserved sequence of the heat shock elements (HSEs) in the promoters of heat-responsive genes (Nover et al., 1996; Scharf et al., 1998; Kotak et al., 2007). As a universal cellular response to a shift up in temperature, the HSF–HSP network is a highly conserved molecular mechanism, representing the first line of inducible defense against imbalances in cellular homeostasis in the prokaryotic and eukaryotic kingdoms. Several lines of evidence support that the heat-responsive expression of HSP and chaperones can be induced by oxidative stress in plants, which can provide a protective function against oxidative stress. In tomato and rice, H_2_O_2_ induced the expression of mitochondrial *HSP22* and chloroplastic *HSP26*, respectively (Banzet et al., 1998; Lee et al., 2000). In cyanobacteria and *Arabidopsis*, high light and H_2_O_2_, respectively, induced activation of some chaperones, HSP, and HSFs at mRNA levels (Desikan et al., 2001; Hihara et al., 2001). To dissect particular stress responses that are relative to H_2_O_2_ signals, various types of model systems including mutants and transgenic plants altered in the ROS levels are applied to investigate the signaling networks. Using genome-wide analysis of the Arabidopsis catalase deficient mutant, a series of genes encoding specific small HSPs, several transcription factors and candidate regulatory proteins were found to be regulated by H_2_O_2_ (Vandenabeele et al., 2004; Vanderauwera et al., 2005). Subsequently, H_2_O_2_ was reported to play a major signaling role in the activation of HSFs during the early phase of heat stress (Volkov et al., 2006). In Class A Hsfs, HsfA2 is highly inducible at the transcriptional level in response to heat stress and the treatments with H_2_O_2_ and ozone (Nishizawa et al., 2006). Intensive studies suggest a critical role of the mitogen-activated protein kinase (MAPK) in H_2_O_2_-mediated expression of Hsfs, including *HsfA2* under heat stress (Kovtun et al., 2000; Link et al., 2002; Sangwan et al., 2002; Kotak et al., 2007; Saidi et al., 2011). According to existing literatures, it has been assumed that H_2_O_2_ is likely to diffuse freely across the chloroplast envelope to trigger a cytosolic MAPK cascade as a signal (Apel and Hirt, 2004). Significantly, treatments with ascorbate or DPI inhibited the heat-induced expression levels, suggesting that H_2_O_2_ acts as an essential component in the heat stress signaling pathway. Interestingly, heat shock promoter element (HSE) protein-binding complex of high molecular weight rapidly (15 min) formed in extracts of heat-stressed or H_2_O_2_-treated cells, indicating that oxidative stress affects gene expression via HSF activation. As described above, ROS act as molecular signals to activate downstream pathways, resulting in protective effects. So far, several redox-sensitive TFs have been identified since their activities rely on redox changes (Tron et al., 2002; Heine et al., 2004; Shaikhali et al., 2008, 2012). Notably, the heat shock factor HSFA4a can act as a sensor of ROS and function upstream of *ZAT12* and *APX1*, two genes with an HSE in their promoters, suggesting that HSFA4a plays an key role in the ROS-mediated heat stress responses (Miller and Mittler, 2006).

To function as a signaling molecule on a cellular level, H_2_O_2_ has to be able to cross the inner and outer envelopes of the chloroplast and other membranes, yet its polar nature might limit its capacity to diffuse through hydrophobic membranes unassisted. In this context, the question arises as to what extent H_2_O_2_ is able to diffuse out of the compartments where it is generated. H_2_O_2_ can be released from isolated chloroplasts immediately (<1 min) upon illumination, as shown by an increase in resorufin fluorescence and detection of electron paramagnetic resonance signals from H_2_O_2_-derived hydroxyl radicals (Mubarakshina et al., 2010). H_2_O_2_ is thought to be able to diffuse through membranes, possibly through aquaporins (Henzler and Steudle, 2000; Bienert et al., 2007; Bienert and Chaumont, 2014), suggesting that channel-mediated membrane transport allows the fine adjustment of H_2_O_2_ levels in the cytoplasm, intracellular organelles, the apoplast, and the extracellular space, which are essential for it to function as a signal molecule. Using fluorescent probe Amplex red which forms fluorescent products in the reaction with H_2_O_2_, the increased production of hydrogen peroxide under high light conditions was observed within the thylakoid membrane, rather than outside the membranes (Borisova et al., 2012). Actually, only a small proportion of chloroplast H_2_O_2_ may cross the chloroplast envelope and directly propagate a cytosolic signal to the nucleus (Mubarakshina and Ivanov, 2010; Borisova et al., 2012). Alternatively, the redox state of cellular compounds like glutathione and ascorbate could be changed by H_2_O_2_ as an oxidant. Furthermore, H_2_O_2_ could affect the reduction state of the quinone pools in chloroplasts, thereby serving as a signal molecule (Pogson et al., 2008).

**FIGURE 1:**
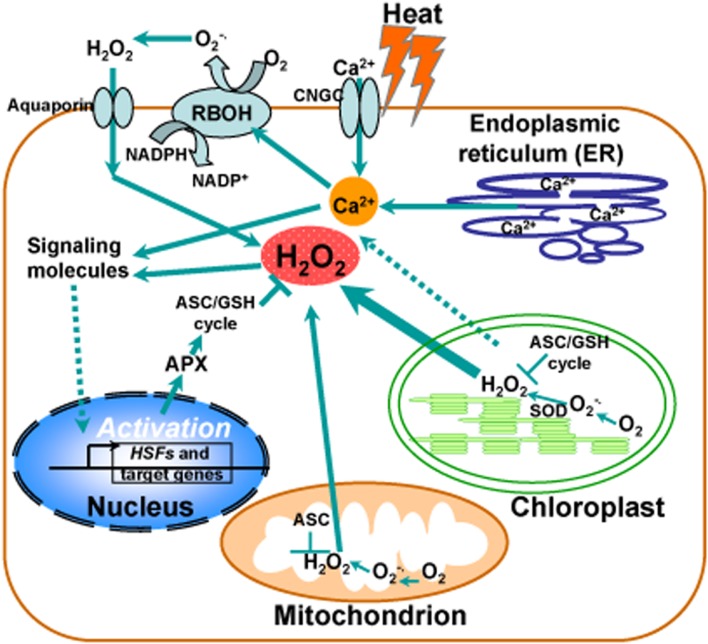
**Calcium and reactive oxygen species (ROS) homeostasis in response to heat stress.** Schematic representation of the major generation sites of ROS and transient calcium increase from different intracellular stores and the influx of extracellular calcium into the cell induced by the opening of cyclic nucleotide gated channels (CNGC) in the plasma membrane in response to heat stress. Heat stress induces activation of calcium channels in ER membranes, leading to the release of calcium in to the cytosol. Chloroplast is a major producer of ROS during photosynthesis under heat stress and contains a large array of ROS-scavenging mechanisms. ROS production also occurs in mitochondria. Hydrogen peroxide (H_2_O_2_) and Ca^2+^ serve as second messengers involved in heat-responsive activation of genes with heat shock elements in their promoters, such as heat shock transcription factors (*HSFs*), heat shock proteins (*HSPs*), and cytosolic ascorbate peroxidase (*APX*). Under heat stress, the maintenance of ROS homeostasis is involved in redox enzymes and metabolites, such as superoxide dismutase (SOD) and the ascorbate–glutathione (ASC–GSH) cycle, functioning in different cell compartments. A NADPH oxidase (respiratory burst oxidase homolog RBOH) in the plasma membrane becomes activated by heat stress via an increased membrane fluidity and/or via a consequent increase in cytosolic levels of Ca^2+^ controlled by a Ca^2+^ permeable channel (CNGC). Ca^2+^ influx activates RBOH by promoting its phosphorylation, leading to the increase of ROS.

Although intensive studies have focused on hydrogen peroxide as a signaling molecule, a clear demonstration of this process is still missing from the literature. Generally, chloroplast is thought to be a major producer of ROS during photosynthesis under heat stress and contains a large array of ROS-scavenging mechanisms whereas ROS production also occurs in mitochondria. Accumulated evidence suggests that H_2_O_2_ and Ca^2+^ function as second messengers to activate the heat-responsive expression of genes with HSEs in their promoters, such as *HSFs*, *HSPs*, and cytosolic ascorbate peroxidase (*APX*), as described in **Figure 1**. Under heat stress, the maintenance of ROS homeostasis depends on redox enzymes and metabolites, including SOD and the ascorbate–glutathione (ASC–GSH) cycle, functioning in different cell compartments. Notably, heat stress induces activation of a NADPH oxidase (respiratory burst oxidase homolog RBOH) in the plasma membrane via an increased membrane fluidity and/or via a consequent increase in cytosolic levels of Ca^2+^ controlled by a Ca^2+^ permeable channel (CNGC). In turn, Ca^2+^ influx activates RBOH by promoting its phosphorylation, leading to the increase of ROS (**Figure 1**).

To date, there is no general agreement on the H_2_O_2_ production rate and concentration in different compartments of the cell (Veljovic-Jovanovic et al., 2002; Queval et al., 2008). Moreover, the molecular mechanisms of H_2_O_2_ diffusion through, however, is still under debate and the necessity for aquaporins for H_2_O_2_ movement *in vivo* is yet to be determined (Foyer and Noctor, 2005, 2013; Pogson et al., 2008; Mittler et al., 2011).

## Possible Cytosolic Components Mediating Plastid-to-Nucleus Signaling in Response to Heat Stress

How retrograde signals are perceived in the cytosol and communicated to the nucleus remains largely unknown. Due to its importance, much research effort has been expended to understand how plants integrate these retrograde signals via a signaling network, which involves second messenger molecules as well as signal-sensing proteins in the cytosol. Thus, we both discuss recent advances in the intermediate signaling components such as calcium ions (Ca^2+^), HSP90 associated complex and PTM between the nucleus and the plastid as well as sensing and signaling cascades in regulation of plant resistance to environmental stresses, particularly to heat stress.

### (i) Ca^2+^, Calmodulin, and Calmodulin-Binding Protein Kinase

Intracellular changes in Ca^2+^ in response to different biotic and abiotic stimuli are detected by various sensor proteins in the plant cell. It is a significant signature that heat shock can trigger a transient increase in [Ca^2+^]_cyt_ in the cytosol of plants (Biyaseheva et al., 1993; Gong et al., 1998; Liu et al., 2003, 2006). Although the molecular mechanisms in regulation of the heat-induced increase in [Ca^2+^]_cyt_ in plant cells remain largely unknown, it is known that the major sources of increased cytosolic free Ca^2+^ are released from intracellular Ca^2+^ pools and extracellular Ca^2+^ stores under heat stress. In *Nicotiana tabacum*, it was observed that heat stress mobilizes cytosolic Ca^2+^ from both intracellular and extracellular sources (Gong et al., 1998). In Arabidopsis, phospholipase C (PLC)/inositol 1,4,5-trisphosphate (IP3) mediates the heat-induced increase in [Ca^2+^]_cyt_ (Liu et al., 2006; Zheng et al., 2012). In the moss *Physcomitrella patens*, it was shown that heat is sensed at the plasma membrane and causes a transient opening of Ca^2+^-permeable channels possibly by modulating the membrane fluidity (Saidi et al., 2009). Using reverse genetic analysis and the whole-cell patch-clamp technique, CNGC6, a member of CNGC family in *Arabidopsis*, was identified as a heat- and cAMP-activated PM Ca^2+^-permeable channel that is involved in heat stress responses (Gao et al., 2012). Interestingly, nitric oxide (NO) acts as a Ca^2+^ upstream signal in heat stress responses (Wang L. et al., 2014). In a recent report, OsANN1 was identified as a Ca^2+^-dependent phospholipid-binding protein, which is involved in heat and drought stress responses by modulating the levels of H_2_O_2_ and redox homeostasis (Qiao et al., 2015). More than 40 putative calcium channels are predicted based on the *Arabidopsis* genome and many of candidate channels have a C-terminus with a putative calmodulin (CaM)-binding domain, suggesting that CaMs are possible components involving the ensuing steps of the heat stress responses (Ward et al., 2009).

Identification and characterization of CaM-modulated proteins in relation to heat stress could prove to be essential for a deeper understanding of the molecular mechanisms involved in heat stress tolerance in plants. It is proposed that the Ca^2+^–CaM complex activates multiple kinases and regulates the activity of heat stress transcription factors in plants (Liu et al., 2003, 2005, 2008; Zhang et al., 2009). For instance, AtCaM3 regulates the expression of *HSPs* by activating calcium/CaM-binding protein kinase (CBK; Liu et al., 2008). These results suggest that CaM may act as an integrator of different stress signaling pathways, allowing plants to maintain homeostasis among different cellular processes (Bokszczanin and Fragkostefanakis, 2013; Virdi et al., 2015). On the other hand, the activation of extensive calcium-dependent protein kinase cascades could provide a mechanism whereby a chloroplast-derived ROS signal would merge into a regulatory network. The transiently increased levels of cytosolic calcium can activate the ROS-producing enzyme RBOHD through the activation of a calcium-dependent protein kinases that phosphorylates RBOHD (Suzuki et al., 2011). The RBOHD-derived ROS can trigger the ROS/redox signaling network that would activate downstream pathways via the important components involving heat stress responses such as MBF1c, certain HSFs and MAPKs (Mittler et al., 2004; Miller et al., 2009). The heat-induced accumulation of ROS and an increase of the cytosolic Ca^2+^ concentration can be sensed by the calmodulin CaM3, which leads to the MPK6-mediated activation of vacuolar processing enzyme that regulates heat-induced PCD (Li et al., 2012).

Not surprisingly, operational control of chloroplast retrograde regulation of heat stress responses is initiated by a combination of factors. In general, Ca^2+^ is a key player in heat stress signal transduction pathways where transient, spiking or oscillatory changes in cytosolic Ca^2+^ levels help to couple environmental cues to appropriate cellular responses. Thus, understanding whether and how much Ca^2+^ signaling contributes to defining stimulus–response specificity has long been a challenge (Monshausen, 2012). Although chloroplasts contain high concentrations (i.e., 4–23 mM) of total Ca^2+^ (Brand and Becker, 1984; Evans et al., 1991), it is not clear that they have the capacity to sequester Ca^2+^ or are involved in the generation of Ca^2+^ signals in response to environmental stresses. The challenge is to design physiologically relevant strategies to define the Ca^2+^-dependent functions of chloroplasts during heat stress responses (**Figure 1**).

### (ii) HSP90-Associated Complex

The 90-kDa HSP (HSP90) is an abundant, evolutionarily conserved molecular chaperone in eukaryotic cells, functioning in the folding and activation of proteins involved in signal transduction, control of the cell cycle and disease resistance (Krishna and Gloor, 2001; Sangster and Queitsch, 2005). In *Arabidopsis*, this HSP90 family includes seven members. The AtHsp90-1 through AtHsp90-4 proteins are classified into the cytoplasmic subfamily, whereas the AtHsp90-5, AtHsp90-6, and AtHsp90-7 proteins are predicted to be chaperones functioning in plastid, mitochondria, and endoplasmic reticulum, respectively (Krishna and Gloor, 2001). It has been suggested that the environmentally sensitive HSP90 complex may initiate signaling cascades in response to environmental perturbations (Sangster and Queitsch, 2005). In a recent proteomic study using an affinity column containing Mg-ProtoIX covalently linked to an Affi-Gel matrix, ligands of the putative signaling metabolite Mg-ProtoIX were identified as three cytosolic heat shock 90-type proteins (HSP90), and interactions between Mg-ProtoIX and were investigated (Kindgren et al., 2011). To test whether the identified interaction between HSP90 and Mg-ProtoIX is biologically relevant, the transgenic lines with repressed *HSP90* levels were generated. Data based on theses transgenic lines suggest that HSP90 proteins respond to the Mg-ProtoIX signal, providing insight into better understanding that how the tetrapyrrole-mediated plastid signal is recognized in the cytosol and further transduced into the nucleus in regulation of nuclear gene expression (Kindgren et al., 2012).

**FIGURE 2:**
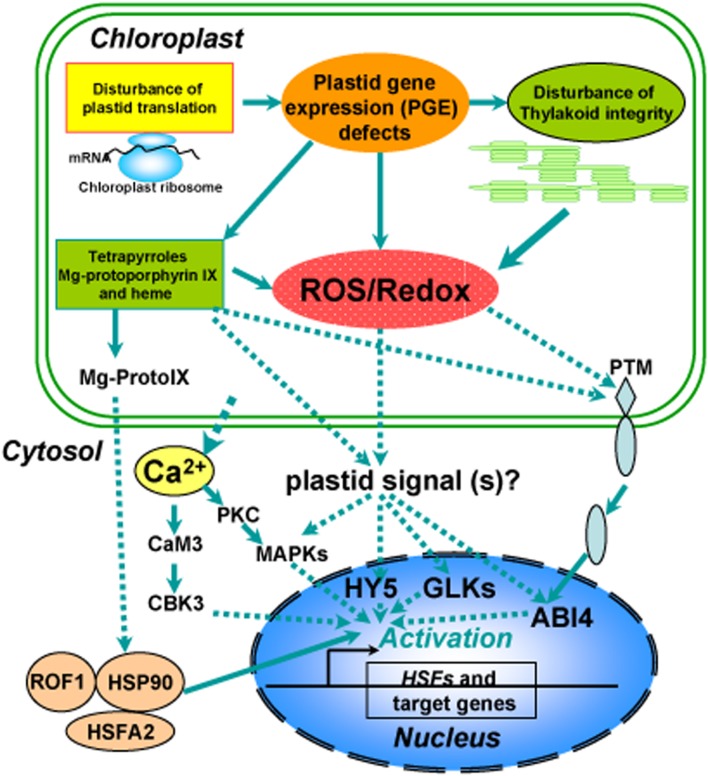
**Overview of a proposed model for chloroplast retrograde regulation of heat stress responses.** Thylakoid membranes are the primary susceptible targets of thermal damage in plants. Heat stress induces disturbance on the photosynthetic machinery and defects in plastid gene expression (PGE), leading to generation of ROS and accumulation of tetrapyrroles such as Mg-protoporphyrin IX (Mg-ProtoIX) and heme. The resulting ROS and disturbance of redox homeostasis in chloroplasts could serve as retrograde signals to activate downstream signal cascades via GLKs, HY5, or ABI4 in the nucleus. Ca^2+^, sequestered from chloroplasts under heat stress, via calmodulin activates calmodulin-binding protein kinase 3 (CBK3) and also activates protein kinase C (PKC), which via phosphorylation regulates MAP kinase (MAPK). The stressed chloroplasts may sequester Mg-ProtoIX, that binds to HSP90, which can form a complex with ROF1 (AtFKBP62), a peptidyl prolyl *cis*/*trans* isomerase. The resulting ROF1-HSP90 complex mobilizes into the nucleus with the help of the heat stress transcription factor HsfA2, which could allow them to drive the transcription of target genes such as *HSPs* required for establishing cellular heat tolerance. A possible retrograde regulatory pathway has also been proposed under heat stress in which PTM, a chloroplast envelope-bound plant homeodomain transcription factor, is cleaved during signaling transduction and the amino-terminal PTM is transferred into the nucleus, where histone modifications to the *ABI4* promoter occur.

Interestingly, ROF1 (AtFKBP62), a peptidyl prolyl *cis*/*trans* isomerase, was shown to be involved in long term acquired thermotolerance by interacting with HSP90.1 and modulating the heat shock transcription factor, *HsfA2* (Meiri and Breiman, 2009). Importantly, heat treatments induce nuclear localization of the ROF1-HSP90.1 complex, which depends on HsfA2 by interacting with HSP90.1 but not with ROF1 (Meiri and Breiman, 2009). These results suggest a role for ROF1 as a cytosolic component in mediating heat stress signal transduction and functions in prolongation of thermotolerance by sustaining the levels of small HSPs that are essential for survival at high temperatures (**Figure 2**). Recently, a direct physical interaction between cytosolic HSP90A/HSP70A and heat shock factor 1 was detected, but surprisingly this interaction persisted after the onset of stress (Schmollinger et al., 2013). It has been accepted that unlike other heat-shock proteins, HSP90 proteins appear not to be involved in protein folding, HSP90 rather interacts with proteins in the near native state and HSP90 is essential for maintaining the activity of numerous signaling proteins (Young et al., 2001; Sangster and Queitsch, 2005).

### (iii) Transcription Factors

So far, very few cytosolic and nuclear components involved in retrograde communication have been identified. The nuclear TF ABI4 (ABA-INSENTIVE 4) belongs to the DREBA3 subgroup of a large family of plant-specific transcription factors known as AP2/EREBP (Sakuma et al., 2002). In a screen for ABA-insensitive (*abi*) mutants, the isolation of ABI4 was the first evidence linking this factor with ABA signaling (Finkelstein et al., 1998). It has been proposed that ABI4 functions as a node of convergence for multiple plastid retrograde signaling pathways in response to GUN1-derived chloroplast signals (Koussevitzky et al., 2007). Moreover, ABI4 binds to this CCAC motif, which has been found to be enriched in promoters of genes that are derepressed during lincomycin treatment in *gun1* and *abi4* seedlings (Koussevitzky et al., 2007). However, its mode of action that relays the chloroplast signals through the cytosol to the nucleus is not fully understood (Leon et al., 2013). Interestingly, treatments with norflurazon and lincomycin induce the high-level production of ^1^O_2_ that has been considered to be a putative signal for the modulation of nuclear-encoded plastid proteins (NEPPs) in response to these inhibitors. However, this regulation is severely inhibited in the mutants of *GUN1* and *ABI4*, indicating that a disruption of tetrapyrrole synthesis may result in localized ROS production or an altered redox state of the plastid, which could mediate retrograde signaling (Moulin et al., 2008). One of the challenging questions is how chloroplast signals are transmitted to the nucleus through the cytosol. The recent identification of PTM, a chloroplast envelope-bound plant homeodomain (PHD) transcription factor with transmembrane domains, potentially links ABI4 and retrograde chloroplast signaling (Sun et al., 2011). Notably, PTM is processed by an unidentified intramembrane peptidase and released from the plastid envelope to the cytoplasm in response to treatments that initiate retrograde signals such as norflurazon, lincomycin, and high light. In turn, the processed PTM transmits into the nucleus, where it directly activates the expression of *ABI4*. Consistence with the role of PTM in retrograde signal transduction, the expression of *ABI4* is much reduced in the *ptm* mutant (Sun et al., 2011). In a recent report, heat-responsive activation of PTM was significantly inhibited in the mutant of plastid *CASEIN KINASE 2* (*CK2*), encoding a major Ser/Thr-specific enzyme regulated by redox signals for protein phosphorylation in the chloroplast stroma (Wang et al., 2014a). These results provide a molecular basis for a chloroplast envelope-bound transcription factor in retrograde chloroplast signaling and shed new light on the mechanism whereby chloroplast signals are transmitted to the nucleus through the cytosol (**Figure 2**). Further experiments focused on identification of the endopeptidase responsible for PTM activation and environmental stimuli that regulate its activity would yield important insight into how the physiological state of the chloroplasts is communicated into the nucleus.

In addition, the transcription factors long hypocotyl 5 (HY5) and Golden 2-like (GLK1/2) are suggested to participate in retrograde signaling pathways and have been shown to respond to plastid signals (Ruckle et al., 2007; Ruckle and Larkin, 2009; Waters et al., 2009). HY5 is converted from a positive to a negative regulator of photosynthesis-associated nuclear genes (PhANG) in response to an unknown plastid signal (Ruckle et al., 2007; Ruckle and Larkin, 2009), demonstrating a convergence between plastid and light signaling networks. A recent study reveals that HY5, together with HSP90 proteins, responds to the tetrapyrrole-mediated plastid signal to control the expression of PhANG during the response to oxidative stress, supporting that Mg-ProtoIX, cytosolic HSP90, and HY5 are all part of the same retrograde signaling pathway that is modified by tetrapyrroles in response to environmental stresses (Kindgren et al., 2012). As members of the GARP superfamily, the GLK proteins are partially redundant and are required for normal chloroplast development (Riechmann et al., 2000). The GLK proteins regulate the expression of genes involved in chlorophyll biosynthesis, light harvesting, and electron transport (Waters et al., 2009). Based on the expression analysis of some retrograde signaling marker genes in the *glk1/glk2* double mutant, the GLK proteins have been implicated in retrograde signaling, specifically in the plastid protein import pathway (Kakizaki et al., 2009; Waters et al., 2009).

Taken together, these findings described above show that the remodeling of chloroplast retrograde signaling networks by these transcription factors is a mechanism by which plants integrate signals describing the functional and developmental state of chloroplasts with signals triggered by a variety of environmental stresses when coordinating the expression of the nuclear and chloroplast genomes during plant adaptation to stresses. These cytosolic mediators can perceive changes of ROS/redox at cellular levels and thus activate rapid and specific responses to environmental cues involving changes in choloroplastic dynamics as well as ROS-dependent signaling networks, although the mechanisms involved remain to be fully established. Chloroplasts can therefore be regarded as a highly important decision-making platform in the cell under heat stress, where ROS and redox play a determining role (**Figure 2**).

## Conclusion and Future Perspectives

The chloroplasts act as sensors of environmental changes and complex networks of plastid signals coordinate cellular activities and function in stress responses to survive environmental perturbations. Therefore, chloroplast retrograde regulation is essential not only for coordinating the gene expression involving photosynthetic processes in both the nucleus and in the chloroplasts but also for mediating plant stress responses. Compared with extensive studies on retrograde regulation of drought and high light stress responses (Fernandez and Strand, 2008; Suzuki et al., 2012; Dietz, 2015; Nagahatenna et al., 2015), our knowledge is very limited in respect to the chloroplast-dependent regulatory mechanisms by which plants respond to heat stress. It is generally accepted that the chloroplasts generates multiple signals in response to a variety of environmental stresses. To respond optimally to environmental stresses, the retrograde signals derived from chloroplasts must transmit the information to the nucleus by cytosolic second messengers or distinct signal cascade pathways. Chloroplasts are metabolically active organelles that used to be regarded as a major source for ROS generated in response to abiotic stress. ROS production can be perceived by the cell as an alarm that triggers stress responses. Although H_2_O_2_ is proposed as a possible retrograde signal molecule, the challenge with this model lies in that how H_2_O_2_ could function as a specific messenger to communicate information on the state of chloroplasts to the nucleus because H_2_O_2_ is generated at different cellular compartments and in response to various different stresses and stimuli in higher plants. In future, identifying the novel interconnecting components of retrograde signaling by innovative genetic or biochemical approaches will significantly advance our understanding of the complicated retrograde pathways in response to a variety of abiotic stresses.

## Author Contributions

F-QG has designed the research topic of this review paper and provided the outline of paper contents; F-QG and A-ZS wrote the manuscript and made the schematic representation figures.

## Conflict of Interest Statement

The authors declare that the research was conducted in the absence of any commercial or financial relationships that could be construed as a potential conflict of interest.
